# Loss of MTAP expression is not an accurate surrogate for CDKN2A homozygous deletions in peritoneal mesothelioma

**DOI:** 10.1111/his.15502

**Published:** 2025-06-25

**Authors:** Andrea Quaranta, Andrea Marzullo, Francesco Fortarezza, Sonia Maniglio, Concetta Caporusso, Floriana Pentimone, Federica Pezzuto, Domenica Cavone, Teresa Lettini, Mario Magistro, Cecilia Salzillo, Luigi Vimercati, Paolo Graziano, Gabriella Serio, Antonio d'Amati

**Affiliations:** ^1^ Section of Anatomical Pathology, Department of Precision and Regenerative Medicine and Ionian Area University of Bari Bari Italy; ^2^ Department of Cardiac, Thoracic, Vascular Sciences and Public Health University of Padova Medical School Padova Italy; ^3^ Section of Occupational Medicine ‘B. Ramazzini’, Department of Interdisciplinary Medicine, Regional Operating Center of Puglia (COR Puglia) University of Bari Bari Italy; ^4^ Department of Radiology, Oncology and Pathology Sciences Sapienza, University of Rome Rome Italy; ^5^ Department of Translational Biomedicine and Neuroscience University of Bari Bari Italy; ^6^ Anatomical Pathology Unit, Fondazione Policlinico Universitario ‘A. Gemelli’ IRCCS Università Cattolica S. Cuore Rome Italy

**Keywords:** biomarkers, *CDKN2A*, CNV, FISH, immunohistochemistry, mesothelioma, MTAP

## Abstract

**Aims:**

Mesothelioma is a malignant neoplasm of the serosal membranes originating from mesothelial cells. Peritoneal mesothelioma is the second most common mesothelial neoplasm after pleural mesothelioma, accounting for approximately 6%–15% of cases. Due to its high morphological variability, often mimicking other lesions, mesothelioma remains a diagnostic challenge. *CDKN2A* homozygous deletion has been established as a highly accurate biomarker for differentiating mesothelioma from benign mesothelial proliferations. MTAP immunohistochemistry (IHC) has been proposed as a cheaper and more reproducible surrogate for *CDKN2A* homozygous deletion (HD) detected by FISH in pleural mesothelioma. The aim of our study was to evaluate the reliability of MTAP IHC as a surrogate marker for *CDKN2A* HD in peritoneal mesothelioma.

**Methods and results:**

Thirty‐nine FFPE tissue samples of PeM were analysed for *CDKN2A* copy number status by FISH. MTAP IHC was performed using antibody clone 2G4, and cytoplasmic positivity was evaluated using two different cut‐offs (1% and 30%). Agreement between IHC and FISH was assessed using Cohen's Kappa. A ROC curve analysis was performed to evaluate the overall diagnostic performance of MTAP IHC. McNemar's test was used to identify statistically significant discordance between the techniques, and a power analysis was conducted to confirm the adequacy of the sample size. Additionally, 14 benign peritoneal lesions were included as external controls and underwent both FISH and IHC. All control samples showed preserved MTAP expression and no *CDKN2A* deletion. *CDKN2A* HD was detected in 27/39 cases. MTAP loss was observed in 13 cases, while the remaining 26 cases showed variable levels of MTAP positivity (15%–100%; mean: 36.4%; median: 25%). Cohen's Kappa revealed a low, non‐significant concordance between MTAP IHC and *CDKN2A* HD (cut‐off 1%: Kappa = 0.091, *P* = 0.462; cut‐off 30%: Kappa = 0.083, *P* = 0.326). ROC curve analysis (AUC = 0.569) confirmed the poor discriminatory performance of MTAP IHC. McNemar's test showed a statistically significant discordance between MTAP IHC and *CDKN2A* FISH results. Power analysis confirmed that the sample size (*n* = 39) was adequate.

**Conclusions:**

These findings may reflect biological and pathogenetic differences between pleural and peritoneal mesotheliomas. Larger, multicentric studies are needed to validate the diagnostic role of MTAP IHC in peritoneal mesothelioma.

## Graphical abstract

MTAP immunohistochemistry (IHC) was evaluated as a diagnostic tool for peritoneal mesothelioma by comparing it with *CDKN2A* homozygous deletion (HD) detected by FISH. Results showed low concordance between MTAP IHC and *CDKN2A* HD, suggesting MTAP IHC is not reliable for predicting *CDKN2A* HD in peritoneal mesothelioma. Further studies are needed.

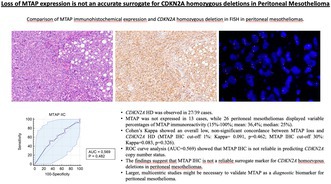

**Abbreviations**
AUC: Area Under the Curve
CDKN2A: Cyclin Dependent Kinase Inhibitor 2A
DAPI: 4′,6‐Diamidino‐2‐phenylindole
FFPE: Formalin‐Fixed Paraffin‐Embedded
FISH: Fluorescence In Situ Hybridization
H&E: Hematoxylin and Eosin
HD: Homozygous Deletion
HPF: High‐Power Field
IHC: Immunohistochemistry
MTAP: Methylthioadenosine Phosphorylase
PeM: Peritoneal Mesothelioma
PM: Pleural Mesothelioma
pRB: Retinoblastoma Protein
ROC: Receiver Operating Characteristic
SSC: Standard Saline Citrate
WHO: World Health Organization

## Introduction

Mesothelioma is a malignant neoplasm arising from mesothelial cells. Although pleural localization is far more frequent, peritoneal origin accounts for around 6%–15% of cases.^1^, ^2^, ^3^

Peritoneal mesothelioma (PeM) most commonly occurs in white population above the age of 60 in industrialized countries. The reported maximum incidence rate, from the UK, is, respectively, 3.6 and 0.7 out of 100,000 people for men and women.^4^

Moreover, genetic predisposition and germline mutations in genes such as *BAP1*, *CDKN2A/B*, *TP53*, *NF2*, *BRCA2*, *ATM* and *APC* have been proposed to play a more prominent role in the pathogenesis of PeM than environmental factors do, in contrast to pleural mesothelioma, which seems to be more linked to environmental carcinogens.^4^, ^5^, ^6^, ^7^

*CDKN2A* homozygous deletion (HD), detected by fluorescent in situ hybridization (FISH), is a reportedly useful biomarker to distinguish between benign mesothelial proliferations and mesothelioma, especially in the pleura.^8^

Deletion is detectable in a high percentage (48% to 61%) of pleural mesotheliomas (PM).^9^, ^10^ It has also been demonstrated as an early event in the pathogenesis of this neoplasm, being already detectable in Mesothelioma in situ,^11^ whereas a lower prevalence of this mutation, ranging from 25% to 35%, is reported in the peritoneum.^2^, ^10^, ^12^, ^13^, ^14^

Furthermore, *CDKN2A* HD has been identified as a possible negative prognostic factor in both PM and PeM.^12^, ^15^, ^16^, ^17^, ^18^

Requiring dedicated equipment and specialized operators, FISH is labour intensive and costly and restricts testing to specialist centres.

Methylthioadenosine Phosphorylase (MTAP) is an enzyme involved in polyamine metabolism, ubiquitously expressed in various human tissues. It is produced by the *MTAP* gene located in 9p21 locus, adjacently to the *CDKN2A* gene, which instead codes for p16 tumour suppressor protein.^12^, ^15^

p16 is a cyclin‐dependent kinase inhibitor, crucial in cell cycle regulation through the inhibition of Retinoblastoma protein (pRB) phosphorylation and subsequent slowing of G1 to S phase progression.^19^

The distance between *CDKN2A* and *MTAP* genes is approximately 100 kb, hence their codeletion is observable in up to 90% of PM cases.^20^, ^21^

Therefore, MTAP immunohistochemistry has been demonstrated to potentially represent a valuable FISH surrogate for the identification of *CDKN2A* HD in PM,^14^, ^21^, ^22^, ^23^ as also specified in the 2021 WHO Classification of Thoracic Tumours.^24^ However, the sensitivity and specificity of immunohistochemical MTAP expression as a surrogate for FISH for *CDKN2A* HD have not been fully investigated in PeM and, consequently, its reliability as a diagnostic and prognostic biomarker for this neoplasm is still rather controversial.

Some studies reported that MTAP loss of expression is highly helpful in differentiating reactive mesothelial hyperplasia from epithelioid PeM in both effusion cytology and surgical specimens.^25^ On the other hand, a recent review by Churg, a new article by Davidson *et al*. and data from the 2023 Update of the Consensus Statement from the International Mesothelioma Interest Group suggest that *CDKN2A* HD and MTAP losses are less prevalent in PeM than in PM, hence possibly playing a marginal role in the peritoneal site.^26^, ^27^, ^28^

The aim of this study is to establish whether immunostaining for MTAP could represent a valuable diagnostic tool for the evaluation of *CDKN2A* copy number status in PeM.

## Materials and Methods

### Ethics Statement

This study was conducted in accordance with the Declaration of Helsinki and approved by the local Ethics Committee of the University Policlinico Hospital of Bari, Italy.

### Cohort Selection

Between 1996 and 2023, fifty (*n* = 50) Formalin‐Fixed Paraffin‐Embedded (FFPE) PeM tissue samples obtained from surgical resections and biopsies were collected at the Policlinico University Hospital of Bari, Italy. The first sample selection criterion was abundance of viable material.

Among the selected cases, male to female sex ratio was 1.8:1, and age ranged from 41 to 89 years old, with a median age of 64, and a mean age of 65.2 ± 10.6.

According to the 2021 WHO Classification of Thoracic Tumours,^24^ purely epithelioid histology was observed in 27 cases (54%) and the remaining 23 (46%) resulted in biphasic (19 cases; 38%) or purely sarcomatous (4 cases; 8%).

To confirm the specificity and sensitivity of our techniques, we included 14 samples of benign mesothelial lesions in our cohort, comprising 8 Nuck cysts and 6 peritoneal inclusion cysts.

### Fluorescent In Situ Hybridization (FISH)

Dual‐colour fluorescent in situ hybridization was performed using the Zytolight SPEC CDKN2A/CEN9 Dual Colour Probe (Zytovision, Bremerhaven, Germany) following the manufacturer's instructions.

Briefly, sections of 4‐μm thickness from FFPE samples were deparaffinized in xylene, then rehydrated and pretreated in 10 mM sodium citrate (pH 6.0) at 80°C for 45 min. After washing in 2x standard saline citrate (SSC) for 5 min, sections were digested with pepsin (37.500 U in 0.1 N HCl) (Sigma‐Aldrich) at 37°C for 10 min and washed in 2 x SSC. Slides were denatured for 16 min at 79°C and then hybridized with the probes and washed according to the manufacturer's protocol. Sections were counterstained using 4,6‐Diamidino‐2‐phenylindole (DAPI) (Vysis, Downers Grove, IL, USA) in fluorguard (Sigma‐Aldrich).

A minimum of 100 non‐overlapped interphase nuclei of consecutive tumour cells in at least two different areas of the section were assessed. *CDKN2A* copy number was considered abnormal when more than 20% of the nuclei had lost both *CDKN2A* signals, but showed at least one CEP9 (homozygous deletion, as suggested by Marshall *et al*.).^29^

Fluorescent analysis was performed on Leica DM6000B fluorescence microscope (Leica Microsystems, Germany) equipped with appropriate filters for DAPI, Orange and green signal visualization.

Any sample lacking centromeric signals or showing excessive epifluorescence phenomena that interfered with result interpretation was discarded.

39 cases (24 (61.5%) epithelioid, 12 (30.7%) and 3 (0.8%) sarcomatous mesotheliomas) and all 14 external controls met the established quality criteria for FISH analysis and therefore underwent immunohistochemical analysis.

### Immunohistochemistry (IHC)

Immunohistochemical analyses were performed by anti‐MTAP monoclonal antibody (clone 2G4 from Abnova Corp., Taipei City, Taiwan) at a 1:200 dilution on 4‐micron‐thick paraffin sections.

Non‐mesothelial immunoreactive cells (lymphocytes, endothelial cells, fibroblasts) were used as internal positive controls.

Immunohistochemical evaluation was performed on all 39 mesothelioma samples and 14 control samples that met the quality criteria for the cytogenetic analysis.

In general, cytoplasmic labelling in tumour cells indicates preserved MTAP expression, whereas the lack of cytoplasmic staining in tumour cells indicates MTAP loss.^21^ Even though inconsistent, nuclear MTAP staining has been recorded in a small percentage of instances. Independently of nuclear staining, only preserved cytoplasmic reactivity was evaluated as preserved MTAP expression.

Cytoplasmic immunoreactivity was defined ‘strong’ when tumour cells stained more intensely than the internal control; ‘faint’ positivity was assigned to tumour cells stained less intensely than the internal control. Cases without sufficient tumour tissue for evaluation or with failed positive internal control were excluded from further analysis.

After determining the percentage of positive tumour cells per 10 HPFs (400x magnification), two different scoring system were established (see below ‘Statistical analysis’).

Analysis was first performed by GS, AdA and AQ alone on a brightfield single‐headed microscope and then by GS, AdA and AQ together on a multi‐headed brightfield microscope.

To prevent any bias deriving from inter‐operator variability, the reported values are mean values from the four analyses, and interrater consensus was assessed by Fleiss's Kappa (Kappa = 0.91).

### Statistical Analysis

Statistical analysis of symmetric dichotomic categorical variables was performed using IBM SPSS Statistics, version 29.0.1.0.

To convert data about MTAP immunohistochemistry into a dichotomic variable, a value of 0 was assigned to tumours that showed a complete loss of MTAP expression and a value of 1 to tumours that showed positivity in more than 1% of the neoplastic cells.

In accordance with previous similar studies on pleural mesothelioma, such as by Chapel *et al*.^23^ and Hida *et al*.,^30^ the samples that showed ‘faint’ cytoplasmic MTAP positivity (defined as weaker than internal positive control), as well as the samples that showed no MTAP cytoplasmic reactivity, were considered ‘negative’ and, therefore, were assigned a value of 0 and considered as loss of MTAP expression.

As for *CDKN2A* copy number by FISH, a value of 0 has been assigned only to tumours showing a homozygous deletion in more than 20% of the counted tumour cells, and a value of 1 to the other cases.

According to some recent evidence from the International Mesothelioma Interest Group,^24^ adopting 30% IHC positivity as a cut‐off for preserved MTAP expression demonstrated improved specificity and sensitivity for *CDKN2A* in pleural mesothelioma. Therefore, also in line with the work published by *Brcic et al*.,^25^ another dataset was built with a score of 0 (loss of MTAP expression) for samples staining positively for MTAP in <30% of tumour cells, whereas a score of 1 was assigned to tumours showing ≥30% of MTAP positivity (preserved MTAP expression).

Data from the two different scoring systems were then laid out separately on two 2 × 2 contingency tables, and agreement between *CDKN2A* FISH and each MTAP IHC scoring system was assessed by Cohen's Kappa.

Specificity and sensitivity of MTAP expression related to *CDKN2A* copy number variations by FISH were then evaluated for both datasets.

Following the obtained results, a nonparametric receiver operating characteristic (ROC) curve was generated, choosing *CDKN2A* copy number status as the classification variable and the percentage of MTAP‐positive cells in each sample as the variable of interest.

For this purpose, cases showing more than 20% of *CDKN2A* HD were defined ‘diseased’, while all the cases harbouring *CDKN2A* HD under the chosen cut‐off were defined ‘non‐diseased’.

Firstly, this test was used to assess the overall performance of MTAP IHC with clone 2G4 for the identification of *CDKN2A* homozygous deletions and, subsequently, to identify an optimal cut‐off by determining Youden's J index.

The ROC curve was calculated with MedCalc Statistical Software, version 22.023.

To further assess the significance of the differences between the results obtained by the two techniques, we performed McNemar's test. Additionally, a power analysis based on the observed discordance rates was conducted to determine whether our sample size (*n* = 39) was sufficient to detect statistically significant differences between MTAP IHC and *CDKN2A* FISH.

## Results

### Fluorescent In Situ Hybridization

Among 39 cases, 27 (69.23%) exhibited a homozygous deletion of the *CDKN2A* gene in more than 20% of tumour cells, 8 (20.51%) harboured a percentage of heterozygous deletion in over 50% of tumour cells, and 4 (10.26%) had no detectable *CDKN2A* copy number variation.

All 14 control samples showed no *CDKN2A* copy number variation, resulting in the presence of 2 orange and 2 green signals per mesothelial cell.

### Immunohistochemistry

**Figure 1:**
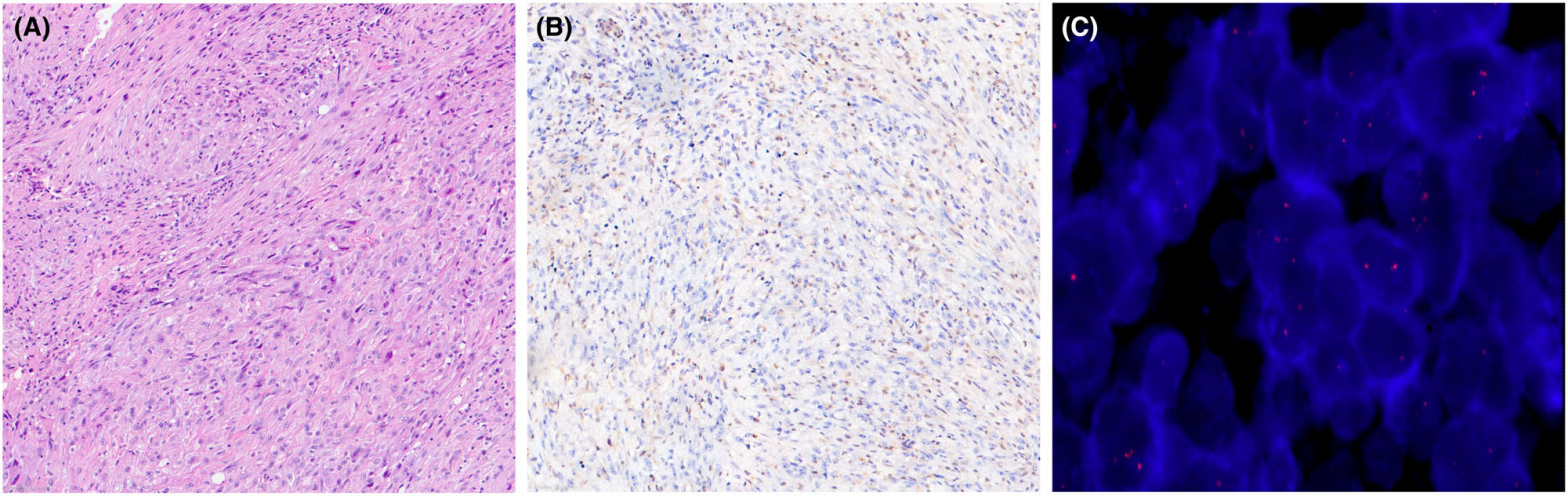
(**A**) Biphasic peritoneal mesothelioma, H&E. (**B**) Loss of expression of MTAP in tumour cells together with immunoreactivity in fibroblastic and inflammatory component as internal control. (**C**) In this case, FISH for CDKN2A did not show a *CDKN2A* homozygous deletion (green signals highlight locus 9p21 whereas orange signals demonstrate the centromeric probe).

Applying the scoring system with the 1% cut‐off, a complete loss of MTAP expression in tumour cells was observed in 13 out of 39 cases (33.33%) (Figure 1). The remaining 26 cases (66.66%) displayed values of immunoreactive tumour cells ranging from 15% to 100%.

Conversely, as regards the analysis conducted applying the 30% cut‐off, preserved MTAP expression was detected in 17 out of 39 cases (43.59%).

All 14 control samples showed a preserved MTAP expression, independently from the applied cut‐off.

**Figure 2:**
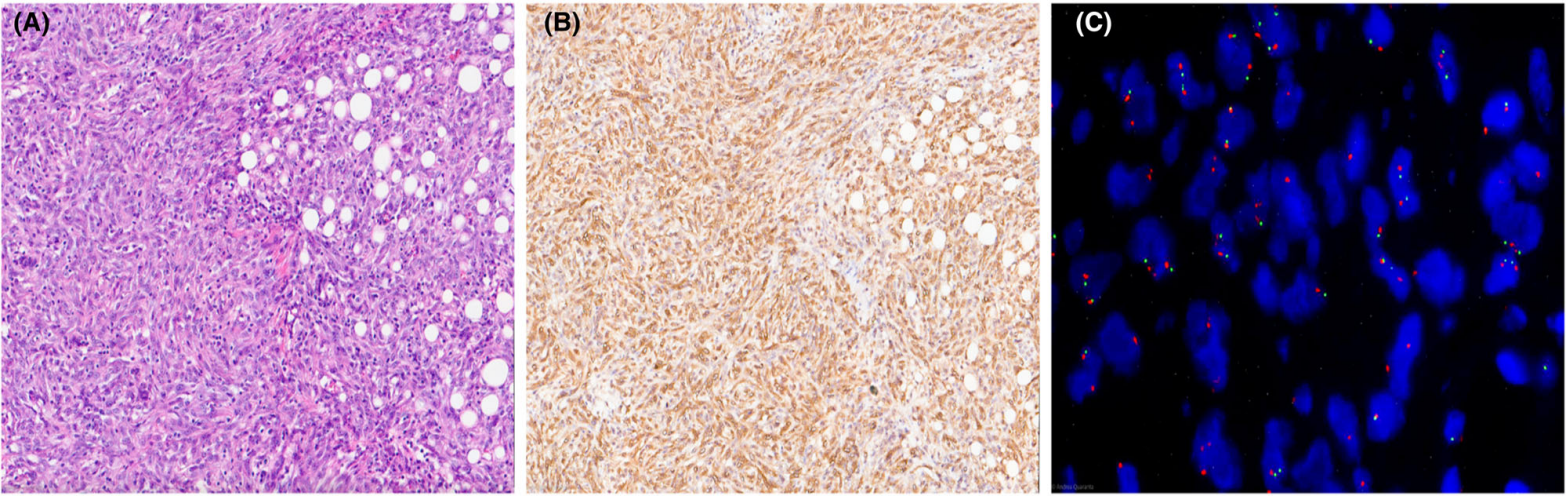
(**A**) Sarcomatous peritoneal mesothelioma, H&E. (**B**) Strong cytoplasmic immunoreactivity for MTAP in all tumour cells. (**C**) In this case, FISH for *CDKN2A* showed homozygous deletion in over 20% of cells (green signals highlight locus 9p21 whereas orange signals demonstrate the centromeric probe).

Interestingly, our analysis also revealed two cases showing a completely preserved MTAP expression (100% of positive tumour cells), even though with a significant rate (>20%) of *CDKN2A* homozygous deletion by FISH (Figure 2). Moreover, 12 cases with high, but not complete, MTAP expression (50%–99% of positive tumour cells) were also found; among them, six cases (50%) harboured a *CDKN2A* HD, notwithstanding preserved MTAP expression.

As previously reported by Chapel *et al*.,^15^ a certain degree of intratumoral heterogeneity in MTAP staining was observed in 7 of our cases (Table 1). In five cases, the heterogeneity involved staining intensity, with strongly positive areas interspersed among weaker ones rather than being spatially segregated. In one case, MTAP staining intensity was stronger in the lower‐grade tubulo‐papillary areas compared to the higher‐grade solid regions. In another case, a biphasic mesothelioma, staining was diffuse in the epithelioid component but markedly weak in the sarcomatoid component.

**Table 1:** Heterogeneity in MTAP staining

Case	Histotype	*CDKN2A* FISH	MTAP IHC positivity	MTAP pattern	Notes
5	Epithelioid	HD	100%	Pattern 1	
6	Biphasic	HD	70%	Pattern 2	
7	Biphasic	HD	50%	Pattern 2	
11	Epithelioid	HD	100%	Pattern 1	
17	Biphasic	1D	60%	Spatial separation	Strong positivity in epithelioid component, loss in sarcomatous component.
20	Epithelioid	1D	80%	Pattern 2	
23	Epithelioid	Normal	75%	Spatial separation	Stronger positivity in lower‐grade areas.

Pattern 1: Strong, diffuse cytoplasmic positivity throughout the sample, with variation in staining intensity; strongly positive cells are interspersed among weaker‐staining cells.

Pattern 2: Strong, diffuse cytoplasmic positivity in the majority of tumour cells, with focal, scattered groups of MTAP‐negative cells interspersed among positive areas.

### Statistical Analysis

The first statistical analysis, based on Cohen's Kappa test (Table 2), revealed a very low level of agreement between MTAP expression and *CDKN2A* homozygous deletion detected by FISH. The results were not statistically significant (Kappa = 0.091, asymptotic standard error = 0.119; 95% CI: 0 to 0.32457; *P* = 0.462). According to this analysis, no statistically significant concordance was observed between MTAP IHC and *CDKN2A* FISH results, indicating a lack of agreement between the two methods.

**Table 2:** With a cut‐off for IHC of ≥1%, MTAP revealed itself to be a rather specific but scarcely sensitive marker for *CDKN2A* copy number status

*CUT‐OFF*	*CDKN2A* HD	CDKN2A normal	Total	
MTAP lost	10	3	13	Specificity: 75%
MTAP retained	17	9	26	Sensitivity: 37.04%
Total	27	12	39	

**Figure 3:**
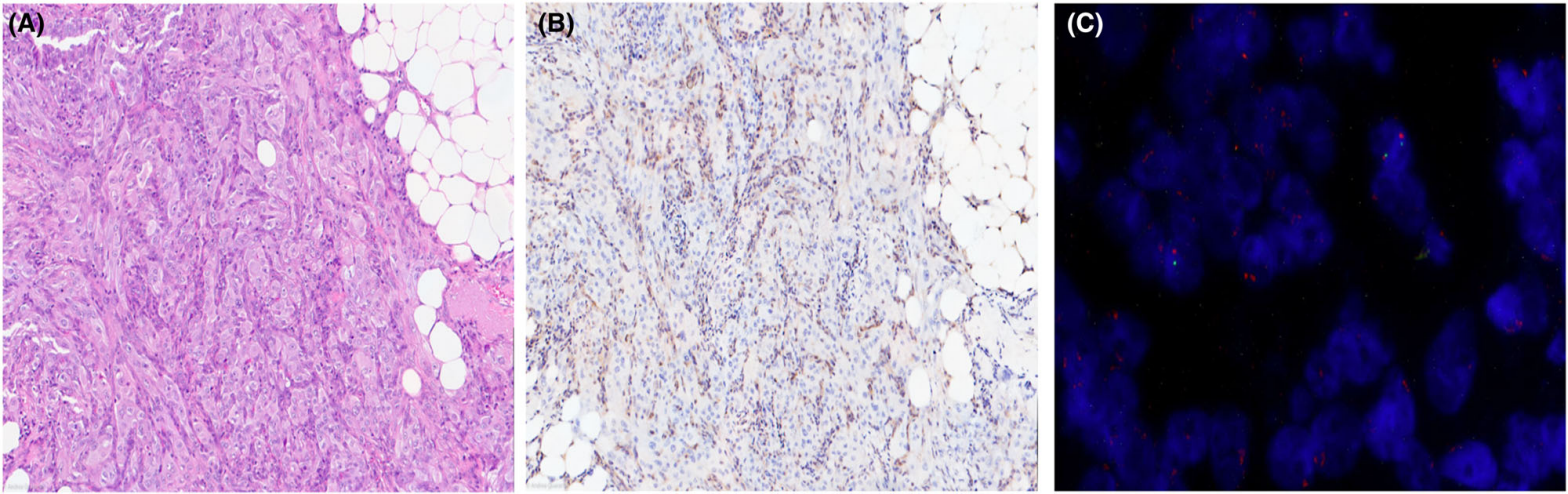
(**A**) Biphasic peritoneal mesothelioma, H&E. (**B**) Loss of expression of MTAP in almost all the neoplastic cells together with immunoreactivity in the fibroblastic and inflammatory component as internal control. (**C**) In this case, FISH for *CDKN2A* showed homozygous deletion in over 20% of cells (green signals highlight locus 9p21 whereas orange signals demonstrate the centromeric probe). [Color figure can be viewed at wileyonlinelibrary.com]

**Figure 4:**
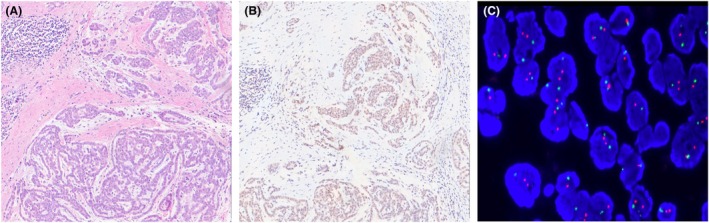
**A**) Epithelioid peritoneal mesothelioma, H&E. (**B**) Preserved expression of MTAP in almost all the neoplastic cells together with immunoreactivity in fibroblastic and inflammatory components as internal control. (**C**) In this case, FISH for *CDKN2A* did not show homozygous deletion (green signals highlight locus 9p21 whereas orange signals demonstrate the centromeric probe). [Color figure can be viewed at wileyonlinelibrary.com]

Among the 27 cases presenting *CDKN2A* HD, only 10 (37.04%) showed complete loss of MTAP expression (Figure 3). Conversely, among the 12 cases lacking *CDKN2A* HD, cytoplasmic expression of MTAP in at least 1% of tumour cells was observed in 9 cases, whereas 3 resulted completely negative (Figure 4).

In light of these numbers, although a complete loss of MTAP expression was a relatively high specific indicator of *CDKN2A* HD (specificity: 75%), it was not highly sensitive (sensitivity: 37.04%).

Applying the 30% cut‐off (Table 3), the concordance between MTAP IHC and *CDKN2A* FISH remains low and not statistically significant (Kappa = 0.083 with an asymptotic standard error of 0.155, 95% CI. −0.22066 to 0.38663, *P* = 0.326) and sensitivity increases to 62% and specificity decreases to 50%.

**Table 3:** Applying a cut‐off of 30%, MTAP IHC remains scarcely informative for *CDKN2A* copy number status

*CUT‐OFF*	*CDKN2A* HD	CDKN2A normal	Total	
MTAP < 30%	16	6	22	Specificity: 50%
MTAP ≥ 30%	11	6	17	Sensitivity: 62%
Total	27	12	39	

**Figure 5:**
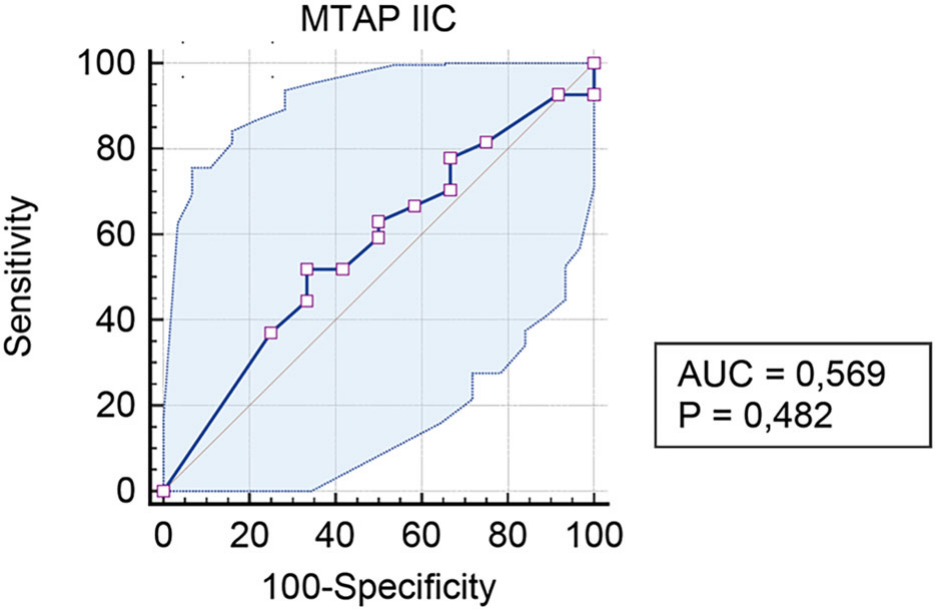
ROC curve analysis. AUC = 0.569 indicates that MTAP IHC has limited predictive value for *CDKN2A* status. [Color figure can be viewed at wileyonlinelibrary.com]

The receiver operating characteristic (ROC) curve (Figure 5) confirmed the results obtained in the first concordance analyses with Cohen's Kappa, proving that MTAP IHC is not informative for the status of *CDKN2A* copy number in our peritoneal mesothelioma cases. Indeed, an area under the curve (AUC) of 0.569 (95% CI. 0.401 to 0.727) is indicative of a poor discrimination ability of MTAP IHC, also suggesting that the observed results are primarily attributable to random chance.

Youden's J index analysis (Youden's index *J* = 0.1852) identified ≤20% MTAP expression as an ‘optimal’ cut‐off for immunohistochemistry, although this value was associated with a rather low specificity and sensitivity (respectively being 66.67% and 51.85%).

McNemar's test revealed a statistically significant difference between the results of MTAP IHC and *CDKN2A* FISH (*χ* ^2^ = 8.45, *P* = 0.0037). Power analysis indicated that the estimated power was 0.92 at *α* = 0.005 (two‐tailed), confirming that our sample size was adequate and the study sufficiently powered to detect statistically significant differences.

## Discussion

PeM is a rare and aggressive neoplasm of the peritoneal serous membranes. Even if prognosis has recently improved,^5^, ^31^ treatment options are still limited,^32^ and an early diagnosis remains of utmost importance to improve clinical outcomes.^23^

Nonetheless, PeM diagnosis is challenging due to its highly variable morphological patterns, often mimicking other entities, and ancillary techniques may often be necessary.^23^

Detection of *CDKN2A* HD by FISH is a *gold‐standard* technique to differentiate mesothelioma from a benign mesothelial proliferation.^5^, ^20^

Aside from its diagnostic value, the presence of a higher rate of *CDKN2A* HD has also been demonstrated to significantly worsen the prognosis in both pleural and peritoneal mesotheliomas.^2^, ^3^, ^12^, ^16^, ^17^, ^22^, ^24^

Along with the 2021 WHO Classification of Thoracic Tumours, recent literature on the topic states that MTAP immunohistochemistry is a highly specific and sensitive surrogate of FISH to detect *CDKN2A* HD in pleural mesothelioma.^14^, ^20^, ^21^, ^23^, ^24^, ^33^

IHC comes with many advantages, such as lower costs, shorter tissue processing times, higher reproducibility, particularly when dealing with higher numbers of samples, and an overall easier interpretation of the results.

Our study represents the first formal evaluation of the reliability of MTAP IHC as a surrogate of *CDKN2A* FISH in a cohort of only peritoneal mesothelioma.

By adopting the same materials and methods as previous studies,^21^, ^22^, ^31^, ^34^ and using a cohort with a histotype distribution comparable to those analysed in other works,^15^, ^23^ our analysis on PeM cases yielded results that differ from those reported by *Chapel et al*. and *Brcic et al*. in pleural mesothelioma,^21^, ^23^ raising concerns about the diagnostic utility of MTAP IHC in peritoneal mesothelioma.

Indeed, our experience demonstrated an appropriate specificity of MTAP IHC when complete loss versus more than 1% of tumour cell expression was observed.

However, sensitivity is lower (approximate value of 37%), meaning that less than half of the cases with *CDKN2A* HD by FISH exhibit a complete loss of MTAP expression. Therefore, confirmation of *CDKN2A* HD by FISH might still be required in routine diagnostic practice.

By contrast, the evaluation of MTAP IHC expression with a 30% cut‐off, as suggested by the International Mesothelioma Interest Group, seems to increase sensitivity despite markedly lowering specificity. Furthermore, both values remain too low (respectively 62% and 50%), suggesting that a FISH confirmation for *CDKN2A* HD would still be recommendable.

ROC curve analysis ultimately demonstrated that MTAP is not an informative biomarker for determining *CDKN2A* copy number status. Additionally, as further support to our findings, the 20% positivity cut‐off value, identified as ‘optimal’ by Youden's J test, failed to ensure high specificity and sensitivity.

A concern regarding this study was whether the lack of statistical significance in some of the results could be attributed to the limited sample size. However, the power analysis we performed for McNemar's test demonstrated that our study was sufficiently powered to detect significant discordance between MTAP IHC and *CDKN2A* FISH. The underlying causes of the heterogeneity observed in MTAP staining remain unclear. One possibility is that different cellular clones within the same tumour may carry distinct genetic and/or epigenetic alterations, which could result in variable MTAP expression—an observation that mirrors the heterogeneity noted in *CDKN2A* deletions by FISH. Additionally, reduced staining intensity might reflect heterozygosity in MTAP copy number, as previously suggested by Chapel *et al*. in 2021.^15^

The discrepancies between results reported in pleural mesotheliomas and in PeM may be linked to many factors. Among the hypotheses, normal *CDKN2A* copy number together with loss expression of MTAP could be related to epigenetic mechanism such as *MTAP* inactivation by promoter hypermethylation, similarly to that described in malignant melanoma by *Behrmann et al*.^35^

Another hypothesis suggests the existence of independent oncogenic and pathophysiological pathways for pleural and peritoneal mesothelioma that might be causative of biological differences between the two forms.

A recent study demonstrated a more frequent loss of expression of MTAP in the pleural site, whereas limited diagnostic value, also unrelated to survival, was reported in PeM.^26^ This data aligns with other studies reporting *CDKN2A* HD to be less prevalent in PeM compared to PM.^27^ Furthermore, there are some alterations, such as *ALK* rearrangements, fusions involving *EWSR1*, losses in loci 1q21 and 8p23, and plausibly germline mutations in genes that are crucial for DNA repair, such as *LIG4* and *ERCC6*, that have been exclusively documented in PeM.^33^, ^36^, ^37^, ^38^

The study we are presenting has however some limitations.

First, this is a retrospective study, nonetheless it involves a relatively large number of cases, also compared to the cohorts selected for similar studies in pleural mesothelioma.^12^ Moreover, all our patients belong to one centre and variability with other centres might always be possible. Different MTAP antibody clones may produce different results as well, as suggested by Febres‐Aldana *et al*.^39^

In conclusion, our study seems to confirm that MTAP has a limited diagnostic value in PeM. Moreover, our data raise doubts regarding the role of MTAP IHC as a possible surrogate for FISH to detect *CDKN2A* HD in PeM, mainly due to its misleading results compared to the actual *CDKN2A* gene copy number status. Larger, multi‐centred studies and, possibly, testing other anti‐MTAP antibody clones, might be useful to further elucidate the role of MTAP IHC in PeM. Overall, our data question the widespread assumption of MTAP as a universal surrogate for *CDKN2A* status across all mesothelioma subtypes, underlining the need for site‐specific validation.

## Author contributions

Conceptualization: A.d.A, G.S., A.Q. and P.G. performed development of methodology and writing, review and revision of the paper; A.Q., A.d.A., C.C. and G.S. provided acquisition, analysis and interpretation of data and statistical analysis; G.S. supervised statistical analysis; A.d.A., A.M., F.F., S.M., F.Pen., F.Pez., D.C., T.L., M.M., C.S., L.V. provided technical and material support. All authors read and approved the final paper.

## Conflict of interest

The authors have no conflict of interest to declare.

## Funding information

The authors did not receive support from any organization for the submitted work.

## Data Availability

The datasets used and/or analysed during the current study are available from the corresponding author on reasonable request.
